# A Novel Flexible Endoscopic C-shaped Suturing Device for Gastrointestinal Wound Closure: First Survival Study in Pigs (With Video)

**DOI:** 10.1055/a-2901-6005

**Published:** 2026-07-14

**Authors:** Zhou-yang Lian, Qiang Zhang, Hong-yan Jin, Xiao-jun Ma, Jie Hu, Zhi Tang, Ran Song, Qi Lin

**Affiliations:** 1 Department of RadiologyGuangdong Provincial People’s Hospital (Guangdong Academy of Medical Sciences), Southern Medical UniversityGuangzhouChina; 2 Guangdong Provincial Key Laboratory of GastroenterologyDepartment of GastroenterologyNanfang Hospital, Southern Medical UniversityGuangzhouGuangdong ProvinceChina; 3 Department of GastroenterologyZengcheng Branch, Nanfang Hospital, Southern Medical UniversityGuangzhouChina; 4 MICRO-TECHNanjingChina

**Keywords:** endoscopy upper GI tract, endoscopic resection (ESD, EMRc, …), endoscopy lower GI tract, endoscopic resection (polypectomy, ESD, EMRc, …)

## Abstract

**Background and study aims** Flexible endoscopic suturing for gastrointestinal wounds is technically challenging and has limited clinical adoption. We developed a flexible endoscopic C-shaped suturing device (E-CSD) and conducted a preliminary pilot study to assess its feasibility and safety.

**Methods** The novel E-CSD was used to close wounds in live pigs following endoscopic full-thickness resection and endoscopic submucosal dissection. Primary outcomes included wound size and location, procedure time, technical success rate, intraoperative and postoperative complications (e.g., device-related mucosal damage, bleeding, and perforation), and wound healing status, including suture maintenance, during a 30-day follow-up period with weekly endoscopy.

**Results** Four wounds were established in two pigs, including two perforated wounds (one each in the stomach and rectum) and two nonperforated wounds (one each in the stomach and colon). The wound sizes ranged from 1.5 × 3.5 to 4.0 × 5.0 cm. Complete closure was achieved in all cases (4/4, 100%), with a mean suturing time of 11 min (range 8–16). No significant complications were reported during the 30-day follow-up period. One case showed minor dehiscence on postoperative day 7, whereas the other wounds remained technically closed and healed progressively into scars.

**Conclusions** The newly developed E-CSD successfully closed both perforated and nonperforated gastrointestinal wounds in live pigs with no significant complications during the 30-day follow-up period. As the first animal study using this method, the findings provide preliminary evidence of the feasibility and safety of E-CSD for gastrointestinal wound closure.

## Introduction

Gastrointestinal endoscopic suturing has emerged as a critical technique for durable wound closure after advanced endoscopic interventions such as endoscopic submucosal dissection (ESD) and endoscopic full-thickness resection (EFR). This approach is a reliable means of achieving secure and durable closure in these complex therapeutic settings. Surgical thread-based techniques under flexible endoscopy yield excellent outcomes. In a study with follow-up through postoperative days 7–21 until scar formation, all sutured mucosal defects remained stable and technically closed without dehiscence. 1

The main flexible endoscopic suturing devices currently used in clinical settings include X-tack, 2 OverStitch, 3 and endoscopic hand-suturing (EHS). 4 5 Among these, X-tack uses nonsurgical thread-based suturing to indirectly fix the thread to the mucosal tissue via screw anchors. In contrast, OverStitch and EHS allow direct suturing without an anchor, and thus more closely resemble surgical suturing. However, operational inefficiency, outer diameter constraints of the device, and the need for frequent repositioning during operation limit the utility of these devices for closure of gastrointestinal wounds.

We developed a novel endoscopic C-shaped suturing device (E-CSD) designed to simplify operation and enhance safety. E-CSD constrains needle motion to a predetermined arcuate trajectory via a circular track, theoretically combining the efficiency of automated devices with the tactile technique of manual suturing. Here, we present the first animal study evaluating the feasibility and safety of E-CSD for closing perforated and nonperforated gastrointestinal wounds. We evaluated technical success, efficiency, and 30-day healing outcomes to provide preliminary evidence for this technology.

## Methods

### Study Design and Animal Models

This single arm trial was conducted on live pigs with pre-prepared gastrointestinal wounds. The study was approved by ClinBridge Biotech Co., Ltd. (Nanjing, China; Approval No.: C250518-20250919) and performed in accordance with institutional guidelines for animal experiments.

Two pigs (mean weight, 60 kg) were used. Bowel preparation was initiated 3 days before the operation and consisted of polyethylene glycol on day 1 and sweetened milk on days 2–3. Animals were fasted on the operation day. Tracheal intubation was performed for inhalation anesthesia during the procedure. Postoperatively, pigs received intramuscular cephalosporin antibiotics for 3–5 days. Oral feeding was resumed on postoperative day 2, beginning with sweetened milk and gradually transitioning to standard feed. All procedures were performed by a single surgeon experienced in ESD and EFR. The operator had no prior clinical exposure to OverStitch or EHS. ESD and EFR were performed to create nonperforated wounds (mucosal and submucosal layers only, with intact muscularis propria) and perforated wounds (full-thickness, transmural), respectively. Wounds were created at the anterior wall of the gastric body near the fundus, the greater curvature of the gastric body, and the colorectum at 15 and 35 cm from the anus. The main endoscopic instruments included a gastroscope (Q260J; Olympus, Tokyo, Japan), single use electrosurgical knife (MK T-2-195-N, MICRO-TECH, Nanjing, China), and the newly developed E-CSD device ( EnClose E-CSD, MICRO-TECH, Nanjing, China).

### Device Features and Operational Essentials

**Fig. 1:**
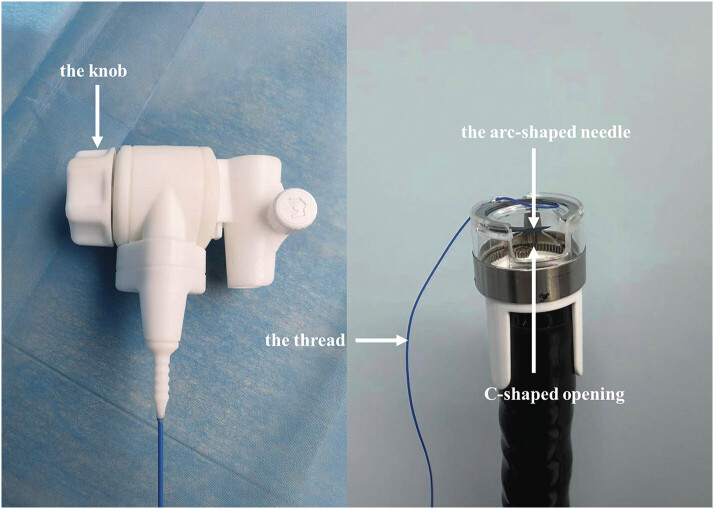
Detailed description of the E-CSD device, including a three-dimensional schematic and photographs. This image presents the knob on the handle that drives an arc-shaped needle, with the head end of the device resembling an endoscope cap, a C-shaped opening, the thread and the arc-shaped needle.

Our E-CSD device comprises the following components ( Fig. 1 ): an arc-shaped needle (housed in a circular track to enable circular motion, with its tail connected to the suture); a suture; a delivery system (to manage arc-shaped needle movement via an integrated handle knob); locking and cutting devices (to enable thread locking-and-cutting); and an optional grasper (for tissue retraction).

**Fig. 2:**
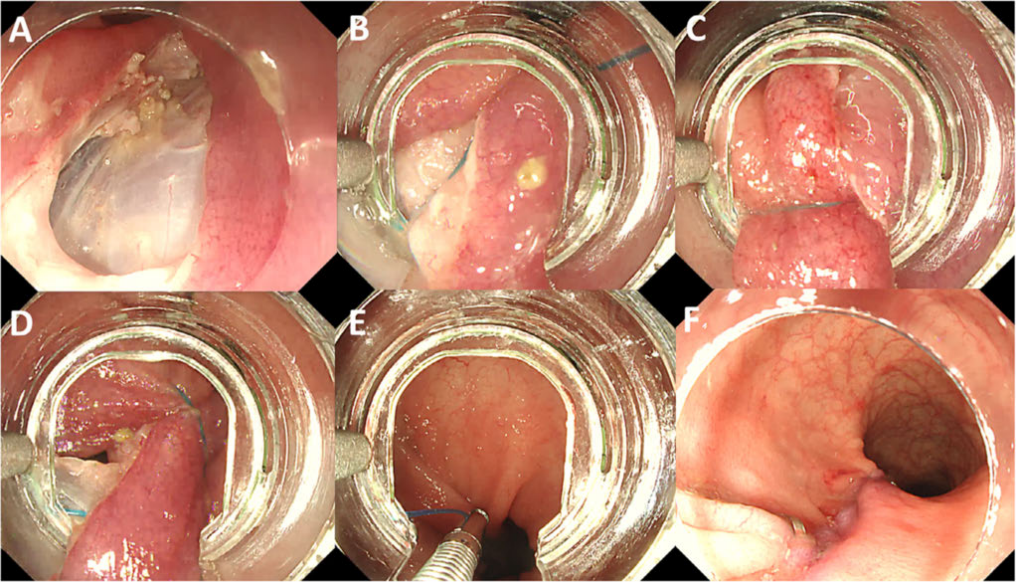
Closure of a rectal perforation wound using the E-CSD. ( **A** ) Establishment of a 1·5 × 3·5 cm perforation wound via EFR. ( **B** ) Compression of the wound edge tissue using the C-shaped opening of the E-CSD device, followed by 360° rotation of the arc-shaped needle using the handle knob for needle threading. ( **C** ) Sequential suturing of the adjacent areas after threading at the first mucosae. ( **D** ) The suture traversed the mucosal tissue at two sites along the wound margin. ( **E** ) Thread tightening, locking, and cutting using the locking and cutting devices device after needle threading at multiple sites. ( **F** ) Successful wound closure with surgical thread.

**Fig. 3:**
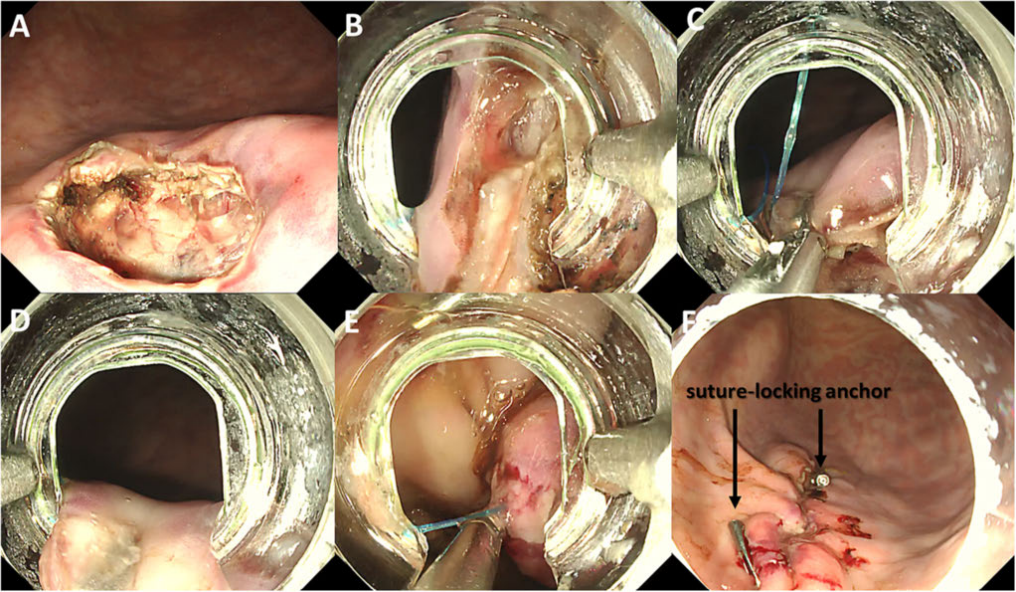
Closure of a perforation in the greater curvature of the gastric body using E-CSD. ( **A** ) Establishment of a 2 × 2·5 cm perforation via EFR. ( **B** ) Compression of the wound edge tissue using the C-shaped opening for needle threading. ( **C** , **D** ) The target tissue was pulled into the C-shaped opening using the grasper for needle threading. ( **E** ) Thread tightening, locking, and cutting using the locking and cutting devices. ( **F** ) Successful wound closure with surgical thread. Two suture-locking anchors were used and left in situ.

During suturing, the device’s C-shaped opening is directly compressed onto the targeted tissue ( Fig. 2B and C ). If the opening cannot be accessed, the grasper is used to grasp the target tissue and retract it into the opening for suturing ( Fig. 3C and D ). The operator then rotates the handle knob to drive the curved needle through a 360° rotation, completing a single needle passage. This step is repeated sequentially along the wound margin, allowing the same suture thread to pass through multiple sites. Next, one end of the suture is secured and cut using the locking and cutting devices and the opposite end is tensioned by the device, secured, and then cut. Finally, two suture-locking anchors are used to lock the suture.

### Outcome Measures

Outcomes were assessed during a 30-day follow-up period with weekly endoscopy. The primary outcomes were wound size and location, procedure time, technical success rate, intraoperative and postoperative complications, and wound healing status. Given the pilot nature of this study, no formal statistical analysis was performed; continuous variables are reported as mean (range) and categorical variables as frequencies (proportions).

Wound location was defined as the distance from the wound to the anus in the large intestine. Procedure time was defined as the interval from device insertion to wound closure. Technical success was defined as complete apposition of wound edges without visible gaps. Intraoperative complications included bleeding, device-related mucosal damage, and perforation, while postoperative complications included bleeding, perforation, and death. During weekly endoscopic follow-up, wound healing status was assessed using the following outcomes: (1) dehiscence of the closed wound; (2) healing with congested, edematous, and irregular mucosa; and (3) complete mucosal healing with scar. At the end of the follow-up period, the pigs were euthanized and dissected. Samples of healed wound tissue were obtained for histopathologic assessment. Additionally, suture stitch count, suture bite, and stitch interval were assessed as key parameters of suturing. A stitch refers to one complete needle passage through both wound margins, bite is the distance from the wound edge to needle entry, and the stitch interval is the distance between adjacent needle entries along the wound margin ( **Supplementary Fig. 1** ).

## Results



**Video 1** Successful closure of a perforated wound in the greater curvature of the gastric body using the new E-CSD suturing device.


**Fig. 4:**
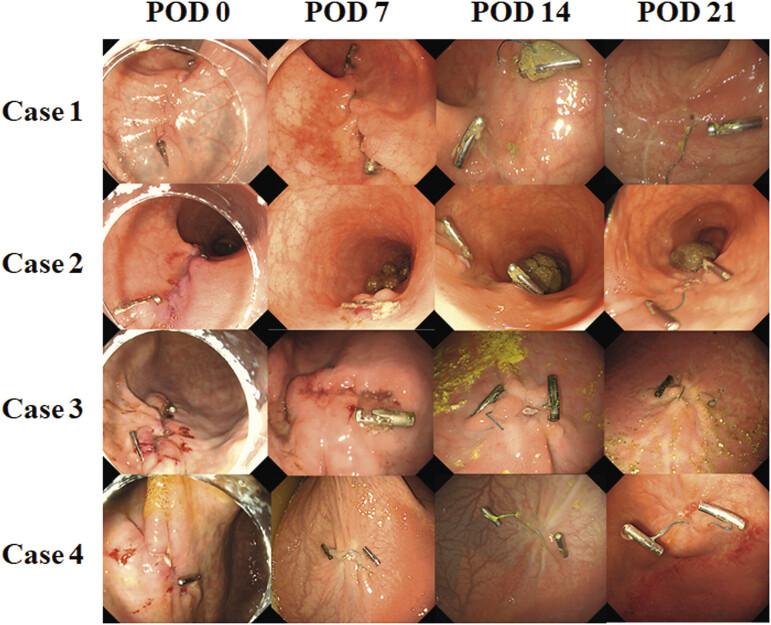
Endoscopic examination was performed to evaluate wound healing after closure. Cases 1–2 represent nonperforated and perforated colorectal wounds, respectively. Cases 3–4 represent perforated and nonperforated gastric wounds, respectively. Postoperative day (POD) 0, 7, 14, and 21 denote the time points of endoscopic examination. In case 2, minor dehiscence was observed on POD 7, and the wound progressively healed into scars. The remaining wounds were closed, mucosal surfaces appeared congested, edematous, and irregular, and progressively healed into scars.

**Fig. 5:**
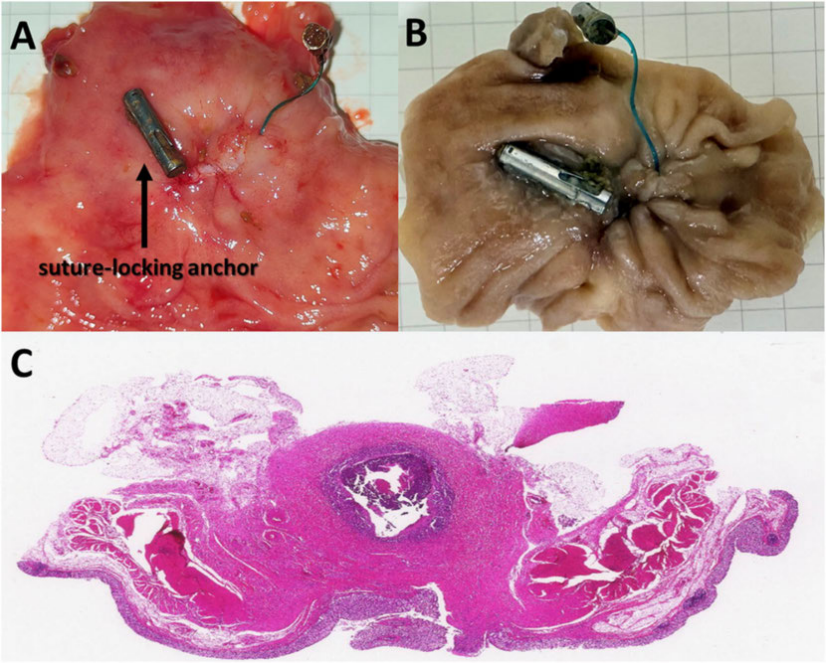
Anatomical specimens of the sutured perforated rectal wound after healing, fixed specimens, and results of histopathologic tissue examination. The two suture-locking anchors remained in situ.

Four wounds were successfully created in two live pigs by EFR and ESD ( Table 1 , Figs. 1 – 4 , Video 1 ), including two perforated wounds (one in the stomach and one in the rectum) and two nonperforated wounds (one in the stomach and one in the colon). The wound sizes ranged from 1.5 × 3.5 to 4.0 × 5.0cm. For E-CSD closure, the suture count ranged from 4 to 7, with a mean suture bite ≥6 mm and a stitch interval of 8–11 mm. Technical success (complete closure) was achieved in all cases (4/4, 100%). The mean suturing time was 11 min (range 8–16 min). No significant complications were observed during the 30-day postoperative follow-up period. Minor dehiscence was observed in case 2 on postoperative day 7. All wounds remained closed, with congested, edematous, and irregular mucosal surfaces, and progressively healed into scars. Anatomic and histopathologic evaluations further confirmed favorable wound healing ( Fig. 5 ).

**Table 1:** Key information in the animal experimental study.

Case	Wound type	Location	Size (cm)	Suture stitch count	Mean stitch interval (mm) ^&^	Suture time (min)	Successful closure of the wound (Y/N)	Postoperative complications (Y/N)/wound closure (Y/N) ^#^			
								POD7*	POD14	POD21	POD30
1	Non-perforation	35 cm from the anus	4 × 5	7	8	10	Y	N/Y	N/Y	N/Y	N/Y
2	Perforation	15 cm from the anus	1.5 × 3.5	4	11	8	Y	N/N ^@^	N/Y	N/Y	N/Y
3	Perforation	greater curvature of gastric body	2 × 2.5	4	8	9	Y	N/Y	N/Y	N/Y	N/Y
4	Nonperforation	anterior wall of gastric body near the fundus	3 × 4	5	10	16	Y	N/Y	N/Y	N/Y	N/Y

#, During the 1-month postoperative follow-up: complications (e.g., bleeding, perforation, and death), and the status of endoscopic wound closure over time (N: dehiscence of the closed wound; Y: healing with congested, edematous and irregular mucosa; complete mucosal healing with scar.). @, In case 2 on POD 7, one minor dehiscence was observed.

*, POD, postoperative day; &, Mean stitch interval = long axis of wound ÷ (number of stitches-1).

## Discussion

The newly developed E-CSD device effectively and safely sutured all wounds in two pigs, and we achieved 100% technical success with no significant complications occurring during the 30-day follow-up period. All wounds healed satisfactorily with scar formation.

X-tack, 2 OverStitch, 3 and EHS 4 5 are the main suture-based flexible endoscopic suturing devices currently used in clinical practice and have demonstrated reliable efficacy and safety for large wound closure. All three devices show high technical and clinical success rates of 93–98% and 93–96%, respectively, with low complication rates—bleeding and perforation occur in 2–3% and 1–3% of cases, respectively. 6 Each of these endoscopic suturing devices has distinct characteristics. X-tack secures sutures indirectly around the wound margin via helical anchors embedded within the tissue. Its helical anchor design makes it particularly advantageous for complex wound closure, including ulcerated wounds, edematous wound edges, and scarred tissues. In contrast, OverStitch, EHS, and the new E-CSD are classic flexible endoscopic suture-based devices. The curved needle must be deployed laterally when using OverStitch, resulting in a relatively large outer diameter. As a result, substantial operating space is needed and applications are limited in confined areas. The E-CSD features a compact distal end, minimizing spatial requirements. EHS has a simple structure and relatively low cost, but involves a longer operation time. 4 5 The new E-CSD and EHS have similar operating principles, both closing wounds by driving an arcuate needle through a 360° rotation for needle passage and thread placement. While EHS requires manual operation, E-CSD adopts a semiautomatic mode that improves efficiency. Moreover, E-CSD’s arcuate needle moves within a fixed circular track and is equipped with a peripheral protective structure, which significantly enhances safety. In the present study, no significant damage occurred to the surrounding mucosal tissue. In this pilot study, E-CSD achieved suturing times of 8–16 min (mean 11 min) for wounds measuring 1.5 × 3.5 cm to 4.0 × 5.0 cm. Historical reports indicated 10–32 min for OverStitch (4.09–4.25 cm wounds) and 33–49.5 min for EHS (2.75–3.6 cm wounds). 6 However, these data are not directly comparable due to substantial heterogeneity in wound size, model protocols, and operator experience.

Sustained closure without dehiscence promotes wound healing. 1 The study showed sustained closure without dehiscence was achieved in EHS-sutured wounds, which contrasts sharply with the 52% mucosal dehiscence rate reported for traditional endoscopic clip closure. 1 7 8 This finding suggests that direct suture passage through wound marginal tissue enhances security. However, sustained closure without dehiscence is also influenced by stitch interval and suture bite. Kobayashi et al. reported dehiscence in all 12 post-ESD gastric defects closed with OverStitch on postoperative day 7. They attributed these results to the wide interval (22·5 mm, 2 stitches/45 mm) and narrow bite (approximately 2 mm). 9 In contrast, a study employing a dense interval (4 mm, 5 stitches/20 mm) and wide bite (7–8 mm) maintained sustained closure without dehiscence until scar formation. ^1^ For our developed E-CSD, the 6 mm C-opening pressed against the wound edge yielded a bite of approximately 6 mm; using endoscopic forceps to pull tissue into the opening can further increase this. We observed partial dehiscence of the closed wound in case 2 on postoperative day 7, which is likely attributable to the relatively wide stitch interval (11 mm), although the remaining wounds exhibited sustained closure without dehiscence.

X-tack, OverStitch, and EHS all employ suture-locking anchors: after placement through wound edge tissue, the sutures are tensioned, anchor-locked, and cut. As a result, dislodgement and expulsion of anchors following wound healing are an issue. For E-CSD, the anchors remained securely fixed at the healed wound sites over the 30-day follow-up period. Although long-term follow-up is warranted to evaluate dislodgement rates, if stability is confirmed, absorbable sutures would permit anchor dislodgement following suture absorption.

This study is subject to several limitations that should be considered when interpreting the results. First, the small sample size (2 pigs, 4 wounds) limits the statistical power and generalizability of our findings. Nevertheless, the promising results justify further assessment of this novel device. The primary endpoints focused on technical feasibility and acute safety, rather than comparative efficacy, which will necessitate larger samples in subsequent studies. Second, as porcine models differ from human physiology, further preclinical evaluation is warranted. Third, the single arm design precludes direct comparison with established alternatives (OverStitch, EHS, or X-tack), limiting definitive conclusions regarding comparative efficacy or superiority. Although our observed suturing times (8–16 min) appear favorable compared with historical controls (OverStitch: 10–32 min; EHS: 33–49·5 min), such cross-study comparisons are constrained by heterogeneity in study protocols, operator experience, and wound characteristics. Randomized controlled trials using standardized wound models are therefore needed to determine whether E-CSD is noninferior or superior to currently used standards. Fourth, the 30-day follow-up period, while sufficient for assessing acute wound healing and early complications, is inadequate for evaluating long-term suture anchor stability. Consequently, dislodgement and expulsion of anchors following complete wound healing remain theoretical concerns. Fifth, our evaluation was limited to the stomach, rectum, and sigmoid colon. Closure of the esophagus, duodenum, and deep colorectal segments present unique technical challenges and will require specific validation. Although preliminary experience suggests potential applicability to these sites, further studies are needed to validate its generalizability. Sixth, the wound tissue specimens were only subjected to routine histological staining, without quantitative assessment of wound healing quality. Seventh, in this study, the device was used to suture the wound on the anterior wall of the gastric body near the cardia, where the distal end of the endoscope was curved but not fully retroflexed. Therefore, device performance under complete retroflexion—such as for gastric fundus wound closure—remains unverified.

Overall, this study provides preliminary evidence of the feasibility of the newly developed E-CSD device for gastrointestinal wound closure in a porcine model, with potential advantages in ease of use, procedural efficiency, and closure reliability. However, further studies with larger samples are warranted to further evaluate its safety and efficacy.

## Supplementary Material

Ergänzendes Material Supplementary Material
