# Supplementary material for: A Novel Flexible Endoscopic C-shaped Suturing Device for Gastrointestinal Wound Closure: First Survival Study in Pigs (With Video)
Source: Endosc Int Open. 2026 Jul 14;14:a29016005. doi: 10.1055/a-2901-6005 (PMC13463268; doi:10.1055/a-2901-6005)

**Supplementary Figure 1. Illustration of the suture bite and stitch interval.**

(A) Pressing the 6 mm C-opening against the wound edge yielded a bite of approximately 6 mm; (B) A schematic illustration.

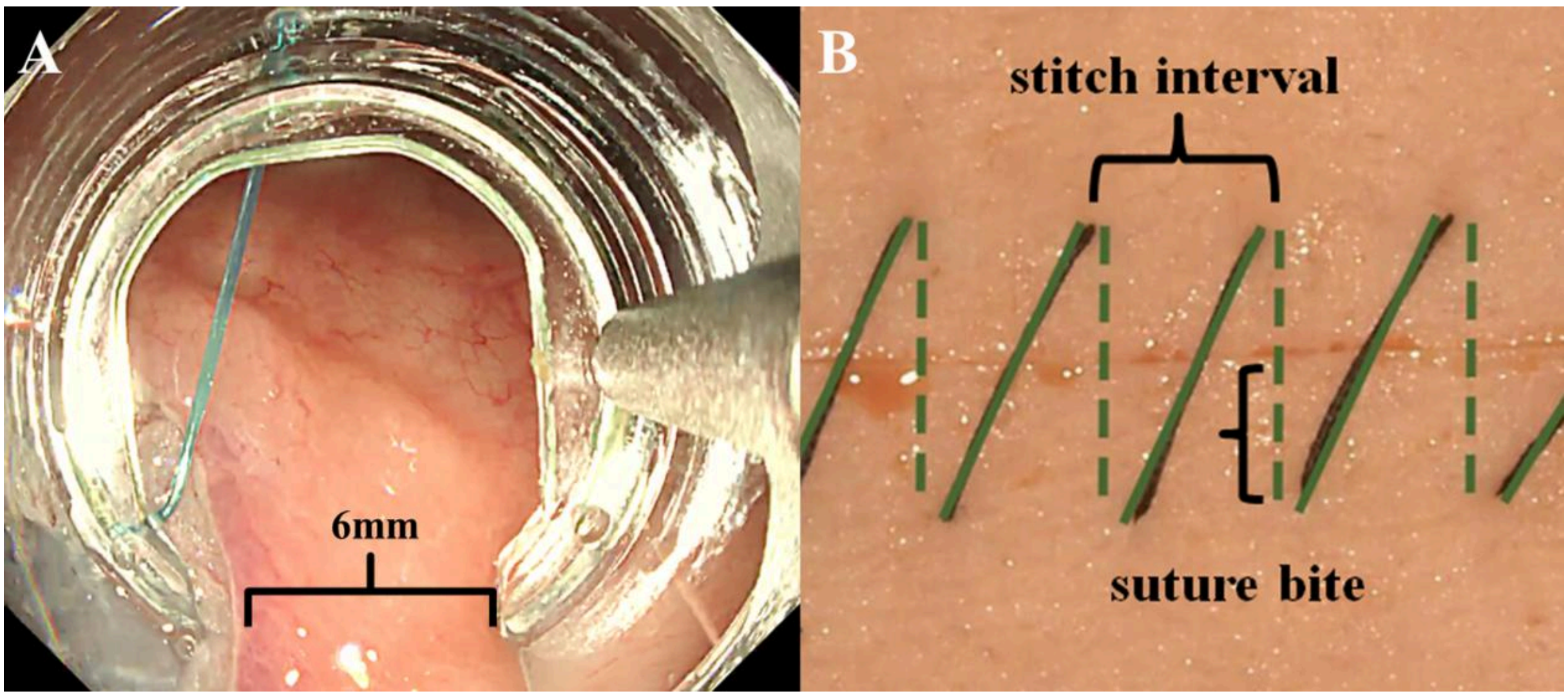

Supplement: Supplementary file 1 [file 10-1055-a-2901-6005_29087503.pdf]
